# Reply to: Reassessing the existence of soft X-ray correlated plasmons

**DOI:** 10.1038/s41467-023-40652-9

**Published:** 2023-10-24

**Authors:** T. J. Whitcher, A. D. Fauzi, C. Diao, X. Chi, A. Syahroni, T. C. Asmara, M. B. H. Breese, A. H. Castro Neto, A. T. S. Wee, M. A. Majidi, A. Rusydi

**Affiliations:** 1 Department of Physics, National University of Singapore, 2 Science Drive 3, Singapore, 117576 Singapore; 2 Singapore Synchrotron Light Source, National University of Singapore, 5 Research Link, Singapore, 117603 Singapore; 3 Centre for Advanced 2D Materials, National University of Singapore, 2 Science Drive 3, Singapore, 117546 Singapore; 4 Department of Physics, University of Indonesia, Depok, 16424 Indonesia; 5 NUS Graduate School for Integrative Sciences and Engineering, Singapore, 117456 Singapore

**Keywords:** Electronic properties and materials, Phase transitions and critical phenomena

**replying to** M. Moazzami Gudarzi & S.H. Aboutalebi. *Nature Communications* 10.1038/s41467-023-39324-5 (2023)

In our recent paper on discovering correlated plasmons in MoS_2_, we used a newly technique combining soft X-ray reflectivity with spectroscopic ellipsometry in a broad energy range^1^. Since then, a recent Matters Arising has brought several important issues to our attention in which we answer within this Response.

The first issue that arose was the difference between our recent data and previously recorded reflectivity and derived complex dielectric functions from earlier sources^2–6^. Whilst the data published between groups has differed over the years, our data are not significantly different from those previous reports. Figure 1a shows the complex dielectric function we measured at 300 K (blue lines) with that of Beal and Hughes (black lines) and although there are differences both the real (solid lines) and imaginary (dashed lines) parts of the complex dielectric function the data are consistent^2^. The main difference is that we have used spectroscopic ellipsometry below 6.5 eV to normalise the reflectivity from 3.5 to 100 eV (and above) and spectroscopic ellipsometry is a self-normalising optical technique^7^, whilst the complex dielectric functions in other publications listed in the Matters Arising are derived from Kramers-Kronig transformation (KKT) of Reflectivity measurements and are not inherently normalised like spectroscopic ellipsometry measurements. The second major difference is that our complex dielectric function has larger values at higher energies than previous reports. This is because we count the spectral weight of higher energy bands up to 45 eV and beyond in our measurements. This spectral weight was not observed from reflectivity within limited photon energy range and also due to the constraint of KKT. In our Supplementary Information of our original publication we point out the differences of using smaller and larger energy ranges to calculate the complex dielectric function, leading to higher values at higher energies, which overall, is a more accurate representation of the material.

**Fig. 1:**
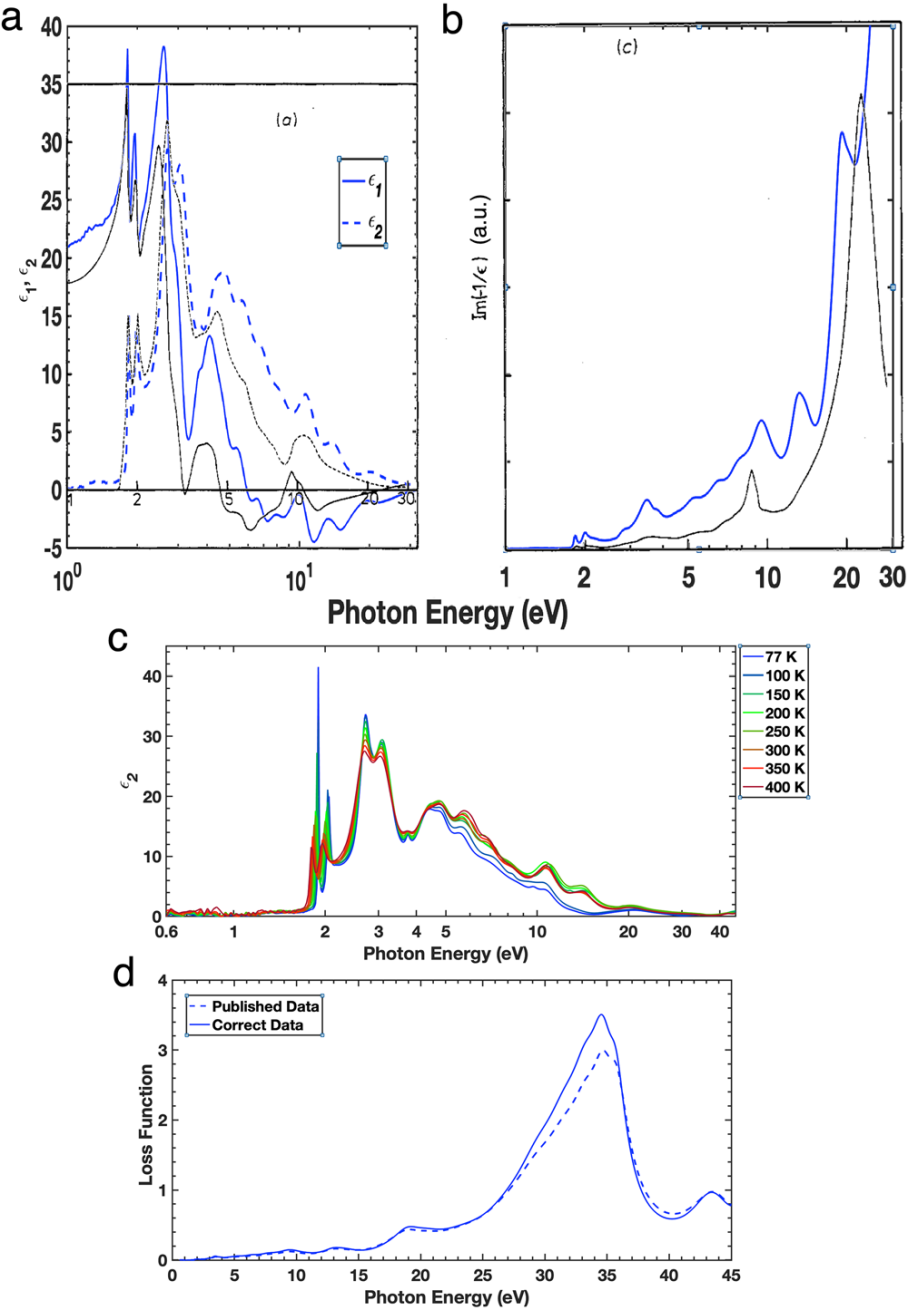
Complex dielectric function of MoS_2_. **a** Comparison of our complex dielectric function measurements of MoS_2_ at 300 K (blue lines) with the Kramers-Kronig transformable data from Beal and Hughes^2^ (black lines). **b** Comparison of our loss function measurements of MoS_2_ at 300 K (blue lines) with the Kramers-Kronig transformable data from Beal and Hughes^2^ (black lines). **c** The imaginary (*ε*_2_) part of the complex dielectric function of MoS_2_ from spectroscopic ellipsometry and soft X-ray reflectance that should have been published as Kramers-Kronig transformable. **d** A comparison of the published and correct loss function data at 300 K.

Figure 1b shows the comparison of the loss function between the 300 K data and the loss function derived by Beal and Hughes^2^. The similarities between the two sets of data is clear along with some differences which we can be addressed. Firstly, it is clear to see plasmon peaks in both sets of data at ~ 9 eV. The difference in energy is small but noticeable. In the Beal and Hughes data^2^, this peak occurs at 8.8 eV, whilst in our data it is at 9.4 eV. This can be explained by the spectral weight transfer from above 30 eV as discussed earlier. We use a larger energy range than in previous publications (up to 45 eV and beyond) and this means that we include higher energy bands. This leads to a shift in the energy of the peak at ~9 eV due to the change in spectral weight. Similarly, there is a difference in the peaks at around 20 eV, in this case 19 eV for our peak and ~21 eV for the peak in the Beal and Hughes data^2^. Again, this difference can be explained by the change in spectral weight due to the larger energy range measured, the much larger and broader 35 eV peak influencing the surrounding spectral weight, and also the much smaller values of *ε*_1_ and *ε*_2_ at higher energies whereby discrepancies are greatly enhanced in the loss function.

The main issue was with the Kramers-Kronig compatibility of our data as demonstrated by calculations in the Matters Arising. This was a valid concern and helped us to find out that the published data for the imaginary part of the complex dielectric function, *ε*_2_, and the loss function were in fact older data. The *ε*_2_ data that should have been published is Kramers-Kronig Transformable and is shown in Fig. 1c, however, the change is small and ultimately has no effect on our main results or conclusions. This is illustrated by the comparison of the loss function between the published 300 K data and the correct 300 K data shown in Fig. 1d. All plasmonic features are still present with only a small adjustment in the magnitude of the loss functions. We adjust the relevant subfigures in Figs. 1–3 in the main text accordingly and will present an erratum in the near future to account for these minor changes in magnitude. These are presented in Supplementary Figs. 1–3.

The authors of the Matters Arising calculated the background dielectric constant, *ε*_*b*_, induced by absorption bands above the cut-off energy of 45 eV, and the number of effective electrons, *N*_eff_, contributing to the optical properties we see within the spectral range of our data. While, these are certainly interesting properties to calculate and give insight into the materials under investigation, great care should be taken when considering the physics. It is well known that MoS_2_ is highly anisotropic material, and certainly within the individual layers it is a highly correlated material, as shown by our XAS data in the original paper^1^. This is famously seen when it is constrained to two dimensions, as monolayer MoS_2_ becomes a direct band gap material^8^. Contrary to the claim in the Matters Arising, one should not treat MoS_2_ as a collection of individual atoms with free electrons of mass *m*_0_, as electrons in highly correlated materials no longer behave as a free electron gas^9–14^ but instead they have an effective mass due to electronic correlation effects^9^ and conventional equations based on free electron gas like those used in the Matters Arising need to be modified accordingly, starting with using *m*_eff_ instead of *m*_0_^11^. Even at room temperature, the effective mass of electrons in crystals is not always equal to the single electron mass, *m*_0_^11^, including in TMDCs^14^.

Nevertheless, for a comparison by taking a free electron picture, in Fig. 2a we recreate the effective number of electrons that contribute to the optical properties of MoS_2_ using the correct values of *ε*_2_, as shown in Fig. 2 of the Matters Arising. Whilst we agree that their assessment of 42 electrons out of 74 per molecular unit is very high, our calculations show that *n*_eff_ is actually much lower, as we obtain numbers similar to their classical calculations of 20.2 using atomic scattering factors as seen in Fig. 2b. Our calculations of the background dielectric constant, *ε*_*b*_, at 45 eV for 300 K using the correct value of *ε*_2_ yield a value of 1.098, which is very similar to the value of 1.043 calculated in the Matters Arising SI, however, our calculated value of *ε*_*b*_ = 0.806 at 45 eV for 77 K is now much lower than their value of 0.92. This is the temperature region where the material becomes highly correlated and thus the physics behind these equations needs to be more carefully evaluated within this new regime and should not be taken classically or trivially, e.g. single or free electron picture. This is also assuming that the effective electron mass, *m*_eff_, is equal to the single electron mass, *m*_0_.

**Fig. 2:**
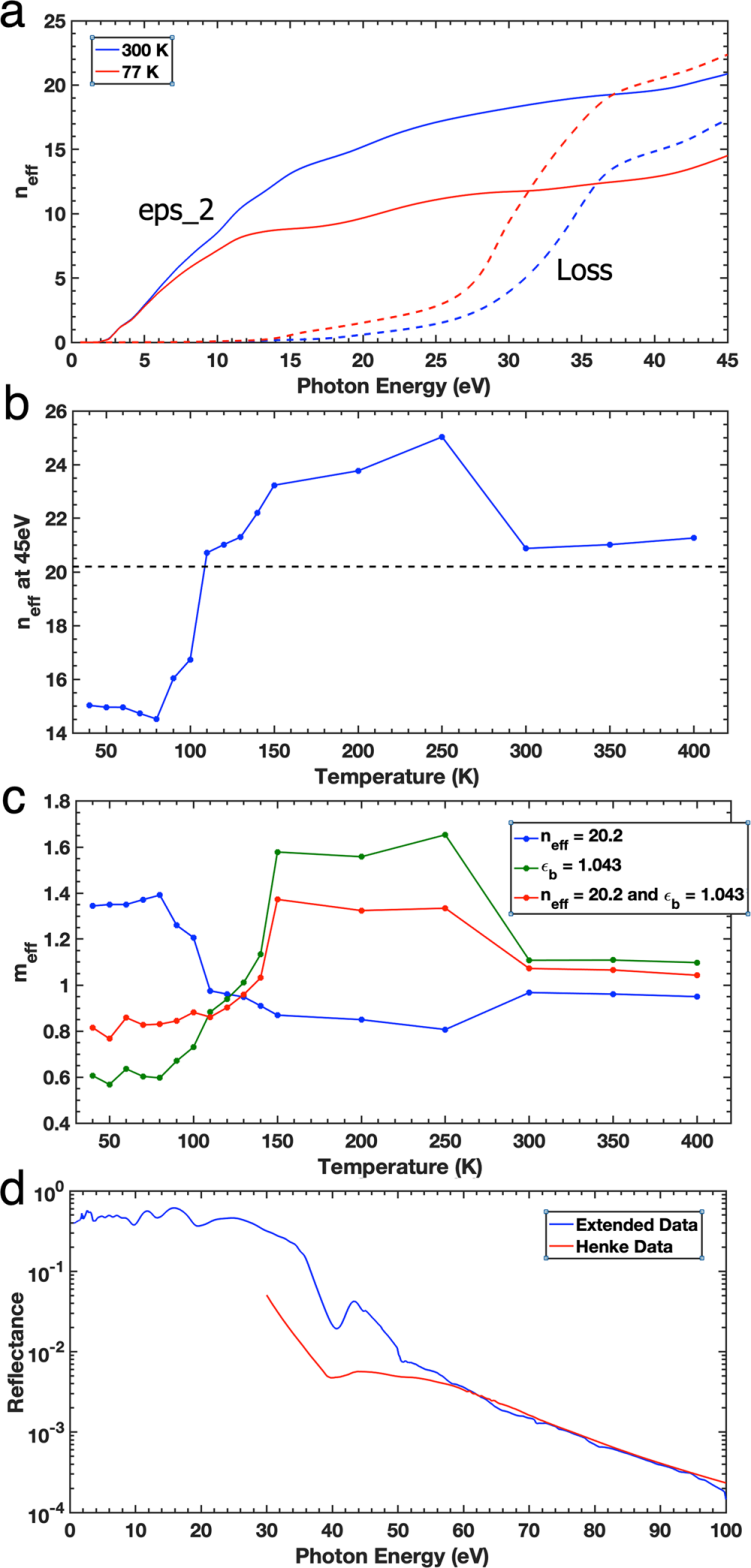
Effective number of electrons in MoS_2_. **a** Our calculations of the evolution of the effective number of electrons (*N*_eff_) contributing to the absorption bands from our 300 K and 77 K data. *N*_eff_ are computed from both *ε*_2_ (solid lines) and the loss function (dashed lines). **b** The *n*_eff_ at 45 eV from our calculations using our data along with the predicted *n*_eff_ of 20.2 shown as the dashed line. **c** The effective mass of electrons as a function of temperature under the constraints of *n*_eff_ = 20.2 and *ε*_*b*_ = 1.043. **d** The high energy normalisation of the MoS_2_ data at 300 K up to 100 eV compared with the Henke data in this range.

As such, using our own data we have assumed the effective mass modifier is 1 and that the effective number of electrons varies with temperature. As at this moment, we do not know the exact number of effective electrons, then the effective mass modifier for our calculations will always be 1 i.e. *m*_eff_ = *m*_0_. Within the Matters Arising, the effective number of electrons at 300 K has been given as 20.2 and the background dielectric constant, *ε*_*b*_, is calculated to be 1.043. Using these numbers, along with our data and Equation1 in the Matters Rising, we calculate that the effective mass, *m*_eff_ is 1.072*m*_0_ at 300 K. Figure 2d shows the results of these calculations across the range of temperatures measured. We have also included calculations of the effective mass of the electrons using *ε*_*b*_ = 1.043 and our measurements of *n*_eff_ shown in Fig. 2b (green line in Fig. 2d) and also using *n*_eff_ = 20.2, and our own measurements of *ε*_*b*_ (blue line in Fig. 2d).

There is a final caveat, in that in all of this we are also assuming that either the effective mass, or the effective number of electrons, changes with temperature. When in reality it is most likely that both of these change with temperature. This would mean that it is more complex than a simple free electron gas picture and we would not be able to tell how either of them would change with temperature using just our results, we would also need data on one of these or the other. In fact, this is an important subject in strongly correlated electron systems and will be the subject of future investigations into this matter. Additionally, it must be clarified that single-particle electronic structure effects such as the hybridisation shown in Fig. 4 of the main manuscript are caused by electron-electron interactions, but do not cause electron correlations, which can be seen in the large spectral weight transfer in Fig. 3 of the main manuscript.

Finally, we address the issue of the high energy normalisation of our data. In the initial publication, we stated that we normalised our reflectivity data to the Henke data above 30 eV and this is indeed correct. The data shown in Fig. 2b of the Matters Arising are also correct in that our normalised data is higher than the Henke data. The reason behind this is that we took reflectivity data up to 100 eV and not just 45 eV as shown in Fig. 2c. As can be seen, our data and the Henke data are normalised within this region^15^. We chose this region because the data within the normalised regime are relatively featureless and we chose 45 eV as our cut-off point because that is where we deemed the most relevant features to our investigation lies. Also, as the Henke data are calculated based on a single electron picture and non-correlated, they do not account for spectral weight transfer in such a broad energy range, and we did not want to lose spectral features from within this understudied spectral region.

In summary, our conclusion on the soft X-ray correlated plasmons stands. We welcome the issues that the Matters Arising has brought to our attention as it has helped us to elaborate on the advanced physics behind highly correlated materials, fix our error in the figures, and to highlight further avenues of investigation within this field. We hope we have settled or answered most, if not all, of the issues presented in the Matters Arising and our result opens discussions on roles of electronic correlation on effective mass of electron, electronic and optical structures in strongly correlated electron systems.

### Supplementary information

Supplementary Information file

## Data Availability

All data that supports the findings of this study are available from the corresponding author upon reasonable request.
