# Supplementary material for: Reply to: Reassessing the existence of soft X-ray correlated plasmons
Source: Nat Commun. 2023 Oct 24;14:6754. doi: 10.1038/s41467-023-40652-9 (PMC10597986; doi:10.1038/s41467-023-40652-9)
Supplement: Supplementary file 1 — Supplementary Information file [file 41467_2023_40652_MOESM1_ESM.pdf]

Reply to: Reassessing the Existence of Soft X-Ray Correlated Plasmons – Supplementary Information

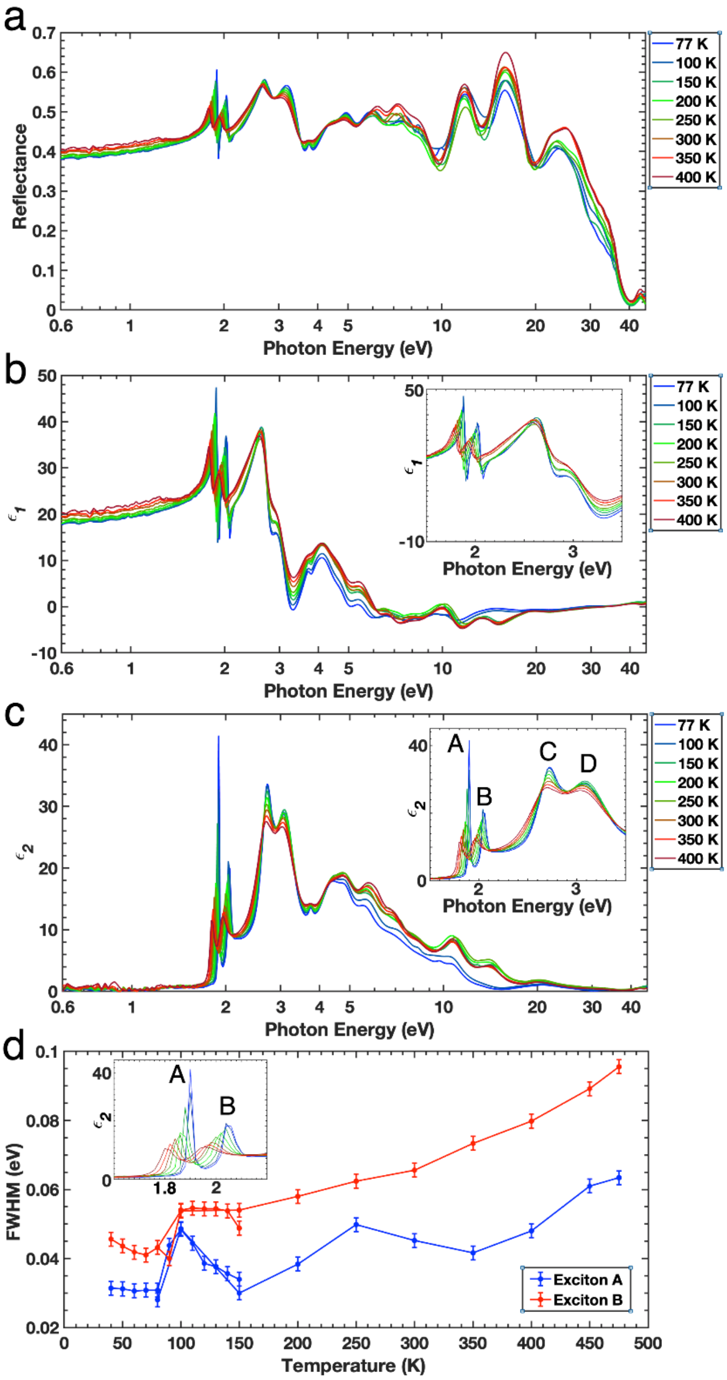

Supplementary Figure 1 | Reflectance measurements and complex dielectric function of MoS_2 as a Function of temperature. **a** Measurements of the reflectance of the bulk MoS_2 from the near-infrared (0.6eV) up to soft X-ray (45eV) using the SUV beamline at SSLS, log scale. The **b** real (ε_1) part and **c** imaginary (ε_2) part of the complex dielectric function of MoS_2 from spectroscopic ellipsometry and soft X-ray reflectance, log scale. **d** The FWHM of excitons A and B as a function of temperature showing significant change. Error bars are

calculated using a 2meV shift. Insets: Magnified view of complex dielectric function of low-energy excitons from **b** and **c** on a linear scale.

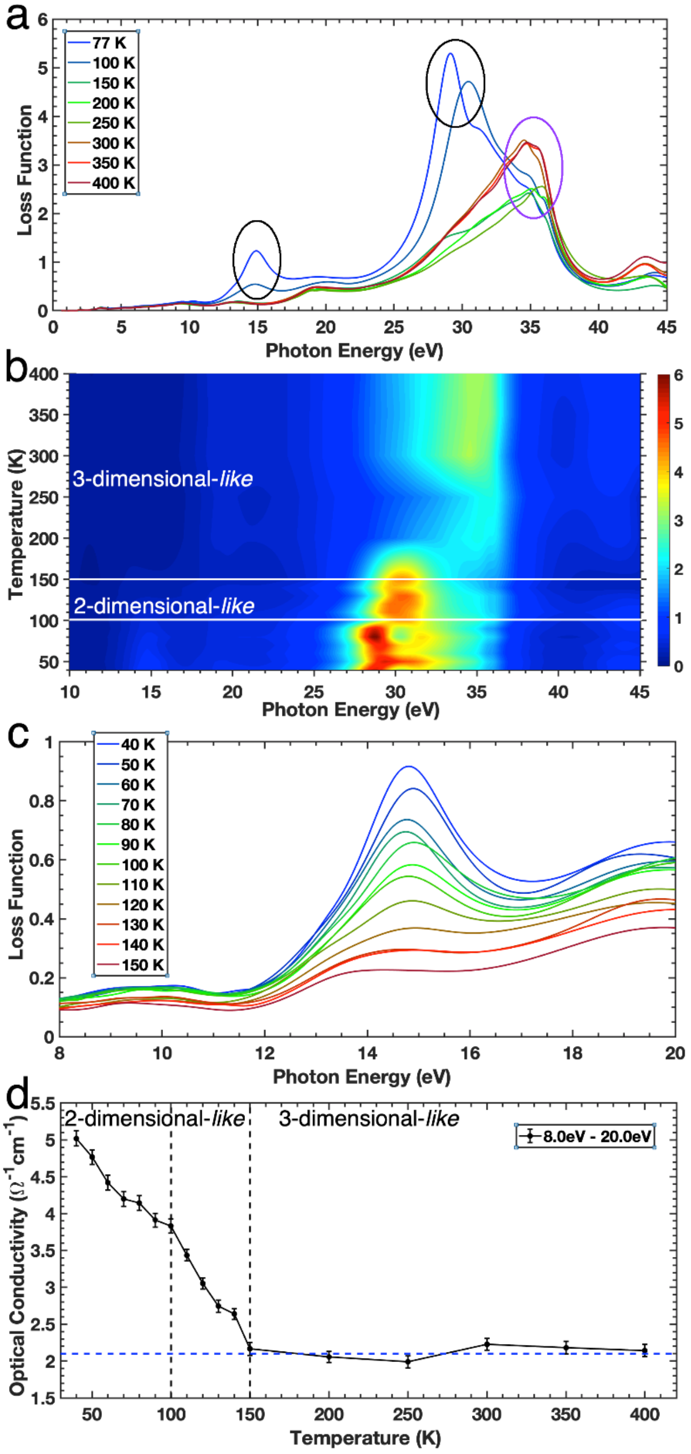

Supplementary Figure 2 | Loss function and spectral weight of bulk MoS_2. **a** The loss function of MoS_2 as a function of temperature from 0.6eV – 45eV. The soft X-ray correlated-plasmonic peaks are circled, i.e. black and purple circles are for low-temperature 2-Dimensional-like and high-temperature 3-Dimensional-like soft X-ray correlated-plasmons, respectively. **b** Contour plot of loss function shows transitions at 150 K and 100 K. Note that vertical dashed lines represent the transition temperatures. **c** Detailed measurements of the loss function of MoS_2 from 8 eV – 20 eV in the temperature range of 40K – 150K at 10K intervals. **d** Evolution of the soft X-ray correlated-plasmon at ~15 eV as a function of

temperature. Note that the dashed vertical lines represent the transition temperatures and dotted horizontal line is a background level. Error bars are calculated using a 200meV shift.

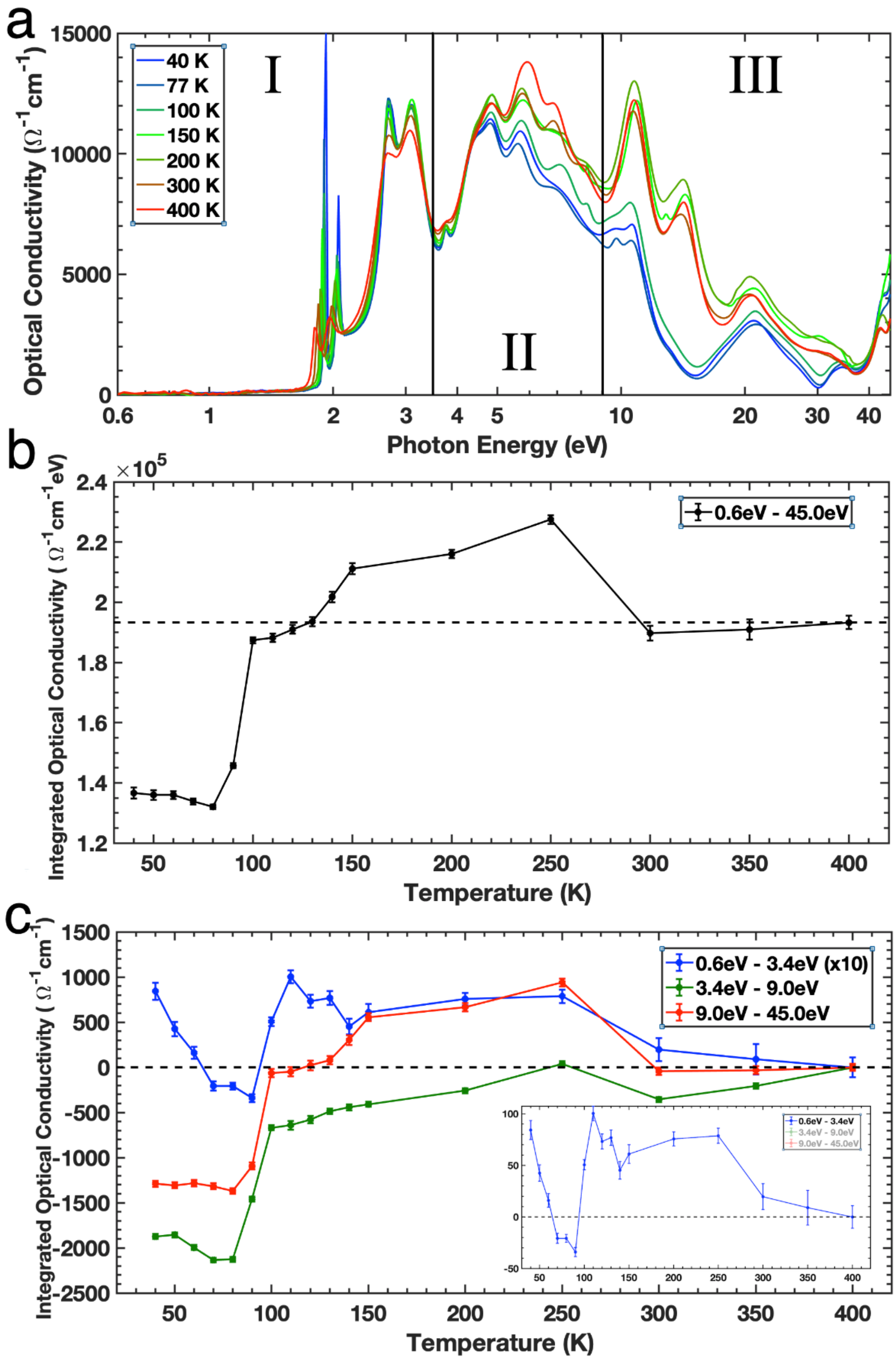

Supplementary Figure 3 | Spectral weight transfer of MoS_2. **a** The optical conductivity of MoS_2 of selected temperatures divided into three spectral regions. **b** The optical conductivity integrated over the whole spectrum as a function of temperature. Error bars are calculated using a 200meV shift of spectral divide. **c** The relative change in integrated optical conductivity of the three different spectral regions as the sample is cooled from 400 K to 40

K. Region I has been magnified for clarity. Inset: Original values of Region I. Error bars are calculated using a 200meV shift of spectral divide.
